# The role of interleukin-15 in the development and treatment of hematological malignancies

**DOI:** 10.3389/fimmu.2023.1141208

**Published:** 2023-04-20

**Authors:** Paola Sindaco, Hritisha Pandey, Colleen Isabelle, Nitin Chakravarti, Jonathan Edward Brammer, Pierluigi Porcu, Anjali Mishra

**Affiliations:** ^1^ Sidney Kimmel Cancer Center, Thomas Jefferson University, Philadelphia, PA, United States; ^2^ Department of Medical Oncology, Thomas Jefferson University, Philadelphia, PA, United States; ^3^ Department of Internal Medicine, The Ohio State University, Columbus, OH, United States; ^4^ Department of Pharmacology, Physiology and Cancer Biology, Thomas Jefferson University, Philadelphia, PA, United States

**Keywords:** IL-15, treatment, blood, cytokine, cancer

## Abstract

Cytokines are a vital component of the immune system that controls the activation and growth of blood cells. However, chronic overexpression of cytokines can trigger cellular events leading to malignant transformation. The cytokine interleukin-15 (IL-15) is of particular interest, which has been shown to contribute to the development and progression of various hematological malignancies. This review will provide an overview of the impact of the immunopathogenic function of IL-15 by studying its role in cell survival, proliferation, inflammation, and treatment resistance. We will also review therapeutic approaches for inhibiting IL-15 in blood cancers.

## Introduction

Cytokines are small, secreted proteins essential for the activation and growth of immune cells and crucial in controlling cell signaling pathways in physiological homeostasis and disease states (1). Released by different types of immune and non-immune cells, they also regulate cell-to-cell communications, with complex regulatory functions in inflammation, cancer, and immunity. Their autocrine, paracrine, and endocrine functions are known to modulate and enhance the anti-tumor immune response and represent exploitable therapies alone or in combination with other agents (2, 3). However, chronic overexpression of cytokines can trigger cellular events leading to hyperproliferation and malignant transformation of immune cells (4). The cytokine interleukin-15 (IL-15) has multifaceted functions and controversial roles in the development and progression of various hematological malignancies but also has a crucial function in the cytotoxic boost against tumoral cells (5). Numerous IL-15-enhancing approaches have been used to treat different cancers, including leukemias and lymphomas, exploiting the main function of the cytokine to potentiate Natural Killer (NK) and T-cell responses and unleash immune-mediated cancer eradication. Nevertheless, the pathogenic role of IL-15 in lymphoproliferative tumors raises concerns about the counterintuitive use of these therapies in this group of malignancies. This review aims to: i. summarize the role of IL-15 deregulation in the pathogenesis of B- and T-cell neoplasms, ii. evaluate its potential as a therapeutic target, and iii. discuss the impact of IL-15 in the context of the new immunotherapeutic agents and adoptive cell therapies.

## IL-15 functions and signaling

IL-15, a 14–15-kD 4-α helix bundle family cytokine member, belongs to the same family as IL-2 and was discovered in 1994 by two laboratories (6–8). It is a pleiotropic cytokine with a major role in the ontogeny, activation, and survival of CD8+ T cells, NKT cells, γδT cells, and NK cells (9, 10). It induces activated B-cells to produce immunoglobulins and is, thus, involved in the interface between nonspecific and acquired immunity (11, 12). IL-15 provides a fast and effective immunology burst to fight invading pathogens. However, compared to IL-2, IL-15 does not affect T-regulatory cells and activation-induced cell death (AICD) (13). IL-15 stimulates cell proliferation and differentiation, works as an anti-apoptotic factor, and its mRNA is expressed by many cell types; however, the protein expression of IL-15 is mainly restricted to activated myeloid cells such as monocytes, macrophages, and dendritic cells (14). Its translation is tightly regulated through multiple negative regulatory elements to limit its potency in eliciting and enhancing the inflammatory response. The large pool of untranslated IL-15 transcripts in different cells can be interpreted as emergency storage, ready to be released in case of a sudden invasion by pathogens or other exogenous insults (15).

**Figure 1:**
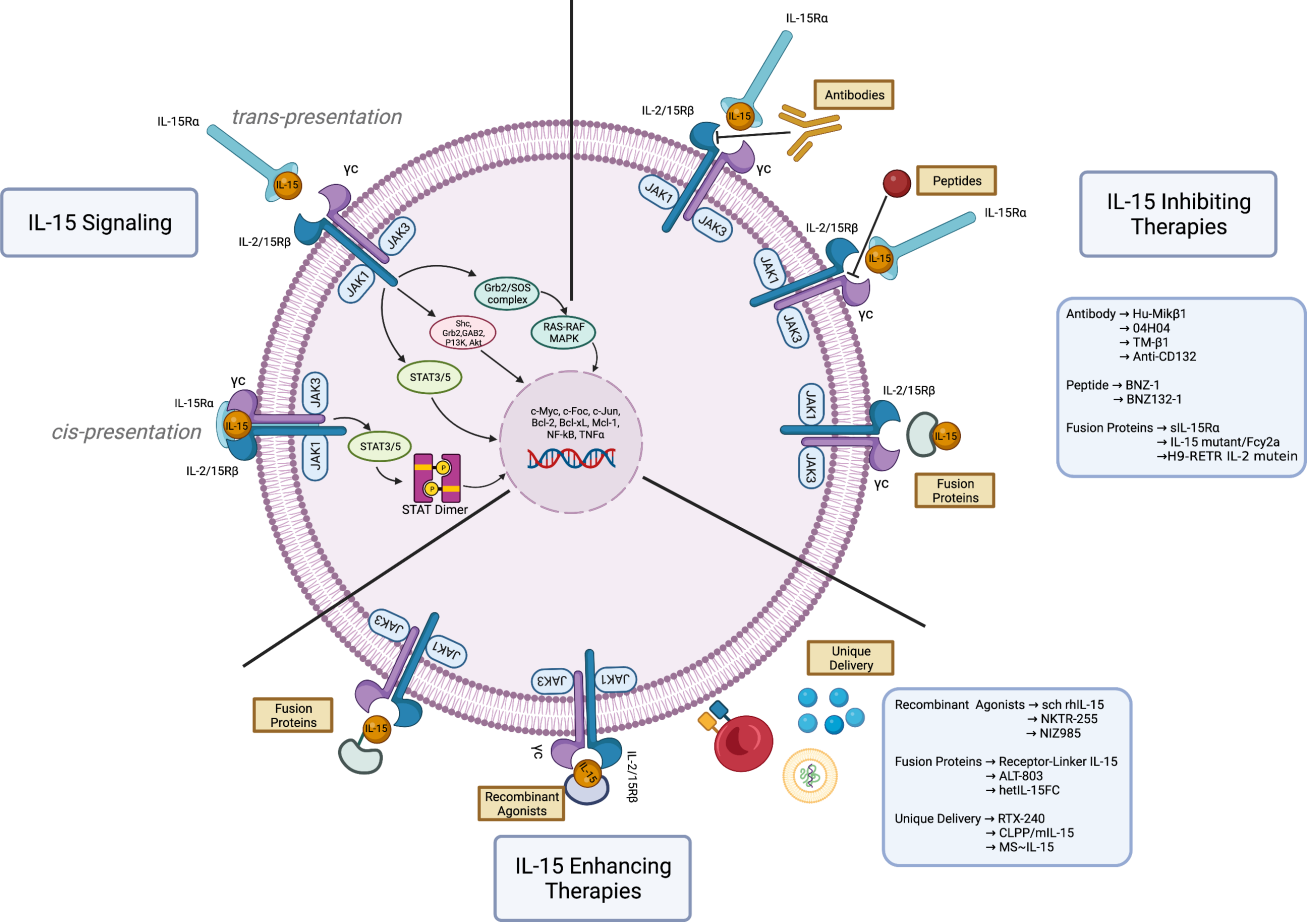
Therapeutic approaches on Interleukin-15 signaling. IL-15 signaling. Interleukin-15 (IL-15) plays a major role in the immune responses by serving as a growth factor for T- and NK-cells. Normal IL-15 signaling begins following the binding of IL-15, produced by dendritic cells, with its high affinity receptor IL-15Rα on the same cells (cis presentation). Upon binding, it is trans-presented to the IL-2R/15Rβ-γc receptor complex expressed by T- and NK-cells, which consequently activates JAK1/JAK3 along with STAT3/5, PI3K/Akt and Ras/Raf/MAPK. These signaling cascades trigger a transcriptional program aimed at boosting the anti-pathogens inflammatory response and enhancing the anti-tumoral immune response. Paradoxically, the overexpression of IL-15 can result in the development of hematological malignancies. IL-15 inhibiting therapies. Numerous interventions have been developed to block IL-15 signaling and the T- and NK-cells tumoral proliferation. Some examples are anti-IL-15 antibody (Hu-Mikβ1, 04H04, TM-β1, anti-CD-132), peptide antagonists (BNZ-1, BNZ132-1), and fusion proteins (soluble IL-15Rα-sIL15Rα, IL-15 mutant/Fcγ2a, H9-RETR IL-2 mutein). IL-15 enhancing therapies. Highly efficient immune response enhancing agents have been developed to leverage the ability of IL-15 to boost the anti-tumoral effector cells. The figure depicts recombinant agonists (sch rhIL-15, NKTR-255, NIZ985), fusion proteins (receptor linker IL-15 or RLI, ALT-803, hetIL-15FC), and unique delivery systems (RTX-240, CLPP/mIL-15, MS~IL-15). These compounds boost the immune response against tumoral cells and can potentiate the effect of immunotherapy and adoptive cell therapy. Figure made with Biorender.com.

The IL-15 receptor consists of a heterotrimeric structure within the plasma membrane, composed of a specific IL-15 receptor alpha subunit (IL-15Rα) (16), a beta subunit (IL-2R/15Rβ) shared with the IL-2 receptor, and a gamma subunit (γc) commonly shared with IL-2, IL-4, IL-7, IL-9, and IL-21 (17). Among the three subunits, IL-15Rα has a greater binding affinity for IL-15 than β and γc subunits; the soluble IL-15/IL15Rα complex is one of the IL-15 functional forms. Soluble and membrane-bound forms of IL-15 have also been described (18, 19). IL-15 can either signal in an autocrine manner by binding to IL-15Rα and IL-2/15R on the same cell (*cis-presentation)*, or paracrine manner that involves presentation of IL-15 bound to IL-15Rα to another cell expressing the IL-2/15Rβγ_c_ transducing receptors (*trans-presentation*) (20–22). IL-15 binding induces conformational changes that lead to activation of the receptor-associated kinases, JAK1 and JAK3, resulting in the phosphorylation of STAT3 *via* the β_c_ and of STAT5 *via* the γ_c_. This causes activation of PI3K/Akt/mTOR and Ras/Raf/MAPK signaling cascades (**Figure 1**), resulting in a proliferative and anti-apoptotic transcriptional program, potentially leading to malignant transformation (23). In addition, PDGFR and IL-15/Akt/XBP1 signaling pathways have been recognized as mediators of the NK cell survival (24, 25). Another recently described mechanism of control from IL-15 on NK cell survival is the trans-endocytosis of the membrane-associated IL-15-IL-15Rα complexes into NK cells, which leads to the activation of STAT5 and subsequent induction of proliferation and survival signals (21). Recently, the IL-15-mediated upregulation of telomerase has been linked to the hyperproliferative program of tumors (26). Due to its complex functions, IL-15 has the potential as a tumor driver and an essential component of the anti-tumor immune response (27).

Various IL-15 transgenic mouse models that carry cloned IL-15 under a variety of different foreign promoters have been instrumental in understanding the role of IL-15 in immunity and blood cancers. Depending on the promoters, genetic background and expression levels, IL-15 mice show either altered immunity or development of aggressive T-/NK- cell leukemia, and cutaneous T-cell lymphoma (13, 28–32). Conversely, IL-15 deficiency has been linked to leukemia development in an immunodeficient mouse model, suggesting a protective role of IL-15 against leukemia (31).

## The pathogenic role of IL-15 in hematological malignancies

Adult T-cell leukemia (ATL), a rare and aggressive leukemia associated with human T-cell lymphotropic virus type 1 (HTLV-1), was the first model that allowed the discovery of some main functions of IL-15 in cancer (6). IL-15 and IL-15Rα mRNAs are overexpressed in T-cells infected with HTLV-1, the viral causative agent of ATL and are related to the proliferation of the malignant cells (14). The viral protein Tax induces the transcription of IL-15 and its receptor through an NF-kB motif (33, 34). Additional findings confirmed that IL-15 is involved in leukemia growth through an autocrine/paracrine mechanism (35). A link between ATL proliferation and the activation of the JAK/STAT pathway, one of the IL-15 downstream pathways, was established (36). The results of a JAK3 inhibitor in *in vitro* and *in vivo* models are promising (37) and clinical trials with drugs targeting the JAK/STAT pathway in ATL are ongoing (NCT01712659).

Cutaneous T-cell lymphoma (CTCL) is a heterogeneous group of lymphoproliferative disorders consisting of aberrant T-cell proliferation in the skin. Numerous studies have demonstrated the overexpression of IL-15 mRNA and protein in CTCL and the level of IL-15 is predictive of the disease severity (32, 38–40). An autocrine/paracrine mechanism has been described for CTCL and IL-15 (39), which allows the tumoral cells to create a self-sustaining microenvironment. IL-15 is responsible for the upregulation of c-myc, c-Jun and Bcl2 in CTCL, through the stimulation of the STAT transcription factors family (41). IL-15 modulates more than 1000 gene transcripts and activates JAK1, JAK3, STAT5, ERK, and PI3K/AKT (42). Another IL-15-dependent mechanism of proliferation in CTCL is the selective γc cytokine-mediated activation of the mTORC1 pathway (43).

Large granular lymphocytic leukemia (LGLL) is characterized by the clonal expansion of large granular lymphocytes (LGL) of both cytotoxic T-cell or NK type in the blood. The abnormal expression of IL-15 and serum IL-15Rα in LGLL is a major player in the pathogenesis of the disease due to their role in regulating T-and NK-cells activation and cytotoxicity, and overexpression of IL-15 in the cellular environment can easily initiate the progression of LGLL, as shown in several studies (44–46). Leukemia raised in IL-15 transgenic mice has been recognized as an *in vivo* model of LGLL, further confirming the cytokine’s pathogenetic role in this disorder (47). IL-15 drives normal large granular lymphocyte *in vitro* and *in vivo* transformation through myc-mediated activation of aurora kinases, DNA hypermethylation and chromosomal instability, leading to cytogenetic aberrancies (44). One of the mechanisms of IL-15-mediated leukemogenesis is the accelerated proteasomal degradation of BID (BH3-Interacting Domain Death Agonist), a pro-apoptotic Bcl-2 family member, that, together with the overexpression of Bcl2 anti-apoptotic genes, leads to the malignant cell aberrant survival (48).

IL-15 can have a role in the pathogenesis of the hematological complication of celiac disease (CD), namely enteropathy-associated T-cell lymphoma (EATL). Mucosa-associated T-cell subpopulations are mainly CD8+ T-cells and can proliferate and expand after an IL-15 stimulus. IL-15 is overexpressed in lamina propria and intestinal epithelium of patients affected by CD and manages intraepithelial lymphocyte homeostasis between their defense role and uncontrolled inflammation and malignant transformation (49, 50).

IL-15 sustains chronic inflammation, autoimmunity, and uncontrolled T-cell proliferation, leading to T-cell lymphoma development (51). The survival signals from IL-15 are mediated by anti-apoptotic factors BCL2/BCL-xL and require IL-15Rβ, JAK3, and STAT5 (52).

As a survival and anti-apoptotic factor for NK cells, IL-15 has a role in the survival and progression of NK cell neoplasms, stimulating tumor growth through the cytokine released by the tumor microenvironment (TME). Monocytes are the main source of IL-15 in NK-neoplasms TME and enhance cell growth by cell contact-dependent interaction *via* membrane-bound IL-15 (53). A large study recognized the prognostic role of serum IL-15 in extranodal NK/T cell lymphoma, but additional studies are needed to understand the impact of the cytokine on the pathogenesis of the disease (54).

Acute lymphoblastic leukemia (ALL) is characterized by the malignant transformation and proliferation of immature B- and T-cells. ALL constitutively expresses IL-15 and γc subunit (55). This expression is higher in the T-cell subtype, in BCR/ABL negative ALL and lymph node and mediastinal infiltration, and, most importantly, seems to have an impact on relapse-free survival (56). Overexpression of IL-15 correlates with disease severity and, more precisely, with Central Nervous System (CNS) involvement, through the upregulation of PSGL-1 and CXCR3, which mediate the homing of malignant cells to the CNS (57, 58), an immunologic sanctuary, where the NK cells are not present (59). IL-15, as a pro-inflammatory cytokine, can contribute to the increased permeability of the blood-brain barrier, facilitating the development of the CNS disease. Although the evidence suggests that IL-15 is associated with ALL development and progression, further studies are needed to identify the molecular mechanisms that are responsible for this observation. The available studies focus on the association between ALL, its clinical features and the level of IL-15 and are more observational. There is a need for more mechanistic investigations aiming to support the involvement of IL-15 in the pathogenesis of the disease.

Several studies have highlighted the importance of IL-15 single nucleotide polymorphisms (SNP) in ALL development, severity, and response to treatment (60–63), with some differences between T-ALL and B-ALL (64). In a cohort of 164 ALL patients, compared to 158 controls, the prevalence of the hetero- and homozygous variants of rs17007695, TC + CC genotype, was 79.6% in ALL patients and 62.7% in controls. The SNPs found in ALL are commonly located within the 3’UTR of the IL-15 gene and can lead to a reduced negative regulatory effect and, thus, an enhanced IL-15 transcription and translation efficiency (61, 65). High IL-15 expression can support ALL development and proliferation and can influence the tumor microenvironment interactions, leading to differences in minimal residual disease and treatment response.

In multiple myeloma (MM), a malignant plasma cell dyscrasia, unbalanced inflammatory cytokines, and strong microenvironment component characterize its pathogenesis. IL-15 and the receptor complex have been identified in MM cell lines and primary MM samples (66). Autocrine IL-15 stimulation is the microenvironment-independent mechanism through which MM cells avoid apoptosis and support their growth. Another study confirmed the role of IL-15 as a key regulator of proliferation and survival in MM (67). In a larger study, serum IL-15 levels were higher in 40 MM patients, compared to healthy controls, with a trend to be overexpressed in advanced stages (68). Further studies are needed to explore the pathogenetic role of IL-15 in MM.

In chronic lymphocytic leukemia (CLL), characterized by increasing accumulation of mature malignant B lymphocytes, IL-15 stimulates cell proliferation *in vitro*, by binding the IL-2R subunits β and γ (69, 70). B-cell proliferation is enhanced by the coexpression of IL-15 and CD40L and is induced by strong activation of STAT5 and ERK1/2 pathways (71). IL-15 can drive leukemogenesis through enhanced mutagenesis due to the suppression of key DNA damage response mediators and overexpression of cyclin D2, *via* PI3K and STAT5 upregulation (72).

IL-15, secreted by follicular dendritic cells, has been recognized in support of germinal center B cell proliferation, pointing out a potential involvement in germinal center-derived B-cell lymphoma pathogenesis (73). The cytokine can be trans-presented by surrounding cells, like macrophages and monocytes, and can promote STAT5-mediated B-cell proliferation and lymphomagenesis (74). In other cases, the monoclonal B-cells can aberrantly secrete the cytokine.

Hodgkin lymphoma (HL), a rare and potentially curable B-cell malignancy, is known to have a highly inflammatory microenvironment and to produce different types of cytokines: IL-15 and its receptor complex are aberrantly expressed, and IL-15 is secreted by the malignant cells and acts as an anti-apoptotic factor in HL and a mitogen through the phosphorylation of STAT5, and ERK1/2 (75). IL-15 is also able to induce resistance to standard chemotherapies. The assessment of IL-15 and IL-15 receptor complex in B-cell Burkitt’s lymphoma lines confirmed the overexpression of the cytokine, mainly in EBV+ cells (76).

IL-15 can have a mitogenic and anti-apoptotic function in selected subsets of acute myeloid leukemia (AML), confirmed by the expression of the IL-2Rβγ complex by blasts (77). Chronic overexposure to IL-15 of NK cells in AML has been recently described: this leads to NK exhaustion and reduced anti-tumor activity and metabolic defects (78). Some of these studies were conducted with longer durations or higher doses of IL-15. These may have affected the outcome of the investigations in different ways, including in animal models and clinical trials. It is important to note that the exposure times and intensity of the IL-15 doses are important factors that can affect the clinical and biological effects of this cytokine.

Additionally, in both primary myelofibrosis (PMF) and myelodysplastic syndromes (MDS), higher levels of IL-15 were detected. but it is only in PMF that it was regarded as a prognostic factor (79, 80). Overexpression of IL-15 in MDS is indicated to cause the growth of memory T-cells in patients (81).

## Therapeutic interventions on IL-15 signaling

The multi-faceted nature of IL-15 encompasses its anti-tumorigenic abilities as a booster of the immune response, and its pathogenic function as a driver of malignant cell proliferation, through the activation of crucial signaling pathways. Both sides of IL-15 function can be exploited in therapeutic approaches against cancer. A list of IL-15-related therapeutic interventions is provided in **Table 1**. Agents that inhibit IL-15 include soluble IL-15Rα, antibodies against IL-15 or components of the heterotrimeric receptor, and steric antagonists of the receptor in the form of a modified IL-2 (**Figure 1**). These approaches showed variable clinical activity and further evaluations of efficacy and toxicity in different hematological malignancies are needed.

**Table 1:** IL-15-related therapeutic interventions: IL-15 is used in hematologic malignancies using anti-IL-15 drugs, IL-15 agonists, chimeric compounds of IL-15 and other molecules, and a combination of IL-15 with adoptive cell therapies.

IL-15 INHIBITORS			
**Anti-IL-15 drugs**	**Structure**	**Functions and clinical trials**	**Ref.**
IL-15α soluble receptor	Soluble form of human IL-15Rα	A potent and specific inhibitor of IL-15, with a high affinity	(82)
Mikβ1	Murine monoclonal antibody against CD122, the beta-subunit shared by the IL-2 and IL-15 receptors	Phase I trial in 12 large granular lymphocytic leukemia patients; no toxicity, no clinical activity	(83, 84)
BNZ-1	Pegylated peptide antagonist that blocks γc receptor to selectively block IL-2, IL-15, and IL-9 signaling	*In vitro* and *in vivo* efficacy in large granular lymphocytic leukemia and adult T cell leukemia cell lines and xenograft models; phase 1 trial showed clinical activity in cutaneous T-cell lymphomas; good therapeutic window	(85, 86) NCT03239392
04H04	Anti-human IL-15 antibody	Used in *in vivo* models of celiac disease with clinical and morphological improvement	(87)
TM-β1	Anti-IL-15 receptor antibody	Used for the chronic inflammation and damage associated with the overexpression of IL-15 in enterocytes and leading to celiac disease enteropathy-associated T-cell lymphoma in a mouse model of the disease	(51, 88)
H9-RETR IL-2 mutein	Engineered modifications of IL-2	Sterically inhibits the binding of IL-2 and IL-15 to the common receptor	(89)
Anti-CD132BNZ132-1	Anti-CD132 (γc subunit)	Specific Inhibitor for the IL-2/IL-15 subfamily; inhibit cell growth in *in vivo* leukemia model	(90)
IL-15 ENHANCING THERAPIES			
**IL-15 agonists**	**Structure**	**Functions and clinical Trials**	**Ref.**
sch rhIL-15	Non-glycosylated monomer, single-chain E. coli-derived IL-15	Increase of NK and effector memory CD8+ T-cells; tumor growth control, short half-life and potential toxicity	(91–94)
hetIL-15/NIZ985	Glycosylated heterodimer of two physiologically active polypeptide chains, IL-15 and IL-15Rα	Extended half-life; robust expansion of NK and CD8+ T-cells; phase I study on unresectable solid cancers: well-tolerated	(18, 95–98)
hetIL-15FC	Fully glycosylated dimeric form in which soluble IL-15Rα is fused to the Fc region region of IgG1	Mimic trans-presentation; increased serum half-life; strong expansion of Natural Killer (NK) and CD8+ T cells	(99)
ALT-803/N-803	IL-15 (IL-15 N72D mutated) and a dimeric IL-15Rα sushi domain-IgG1 Fc fusion protein	35-fold longer serum half-life; wide therapeutic window, low toxicity, five-fold increased biological activity; active in murine B-cell lymphoma, in phase I clinical trial on non-Hodgkin lymphomas, multiple myeloma and acute myeloid leukemia (Overall response rate 19%)	(100–108)
Receptor-Linker-IL-15 (RLI)	L-15Rα sushi domain (N-terminus region) fused to IL-15, via a 20-amino acid flexible linker	Prolonged IL-15 plasma level; strong upregulation of CD69, Bcl2 and Bcl-xl	(109)
NKTR-255	PEG conjugate of recombinant human IL-15 (rhIL-15) receptor agonist	Engages the entire IL-15 receptor complex (IL-15Rα/IL-2Rβγ); enhanced anti-tumor efficacy of human CD19 CAR-T in *in vivo* lymphoma model; ongoing phase I clinical trial of combined CD19 CAR-T and NKTR-255 for R/R B-cell malignancies	(110–114)
Il-15 mRNA	*in vitro* transcript mRNA in a protamine/liposome system (CLPP)	High therapeutic potential of the CLPP/mIL-15 complex for colorectal cancer therapy	(115)
MS~IL-15	Hydrogel microspheres (MS) covalently attached to IL-15 (MS~IL-15) by a releasable linker	Very long-acting IL-15; prolonged expansion of target immune cells and high anticancer activity	(116)
**IL-15 agonists+other molecules**	**Structure**	**Functions and clinical trials**	**Ref.**
N-809	Bifunctional fusion protein IL-15/IL15Rα superagonist complex containing the Fc-domain of IgG1 fused to two single chain anti-PD-L1 domains	Enhanced CD8+ and NK cell cytotoxicity against tumoral cells	(117)
αPD-1-IL-15-R	Engineered anti–PD-1 fusion protein with IL-15-IL- 15Rα	Extraordinary *in vitro* and *in vivo* anti-tumor efficacy with no toxicity	(118)
KD033	Fully human, high- affinity anti-programmed death-ligand 1 (PD-L1) linked to the sushi-domain of the human IL-15/IL-15 receptor alpha complex	Robust anti-tumor effect in *in vivo* models	(119)
**IL-15 and adoptive cell therapy**	**Structure**	**Functions and clinical trials**	**Ref.**
rhIL-15 and haploidentical natural killer cell therapy	Association of adoptive cell therapy and IL-15	42 acute myeloid leukemia patients: 32% achieved complete remission; cytokine release syndrome and neurological toxicities	(120)
anti-CD19 CAR-NK	NK cells transduced with a retroviral vector expressing genes that encode anti-CD19 chimeric antigen receptor, CAR, interleukin-15, and inducible caspase 9 as a safety switch	IL-15 to enhance the *in vivo* expansion and persistence of the transduced NK cells	(121)
CAR-T-IL-15/IL-15Ra	Constructed CD19 specific CAR-T cells overexpressing IL-I5 and IL-15 receptor alpha (IL-15Ra)	IL-15 enhances T cells persistence and IL-15Rα reduces the adverse effects of IL-15. CAR-T cells show more effectiveness	(122)
NKL-IL15	Human interleukin-15 (hIL-15) gene-modified NKL cells	IL-15 gene-modification augment NK cell-mediated anti-human leukemia function	(123)
NKG2C-KE	Anti-NKG2C/IL-15/anti-CD33 killer engager	Efficient engagement of NK cells against tumoral cells; high efficacy in *in vitro* model of acute myeloid leukemia;	(124)
32Dp210-IL-15/IL-15Ra/CD80	IL-15/IL-15Ra/CD80-expressing AML cell as post-remission vaccine	50% overall survival in *in vivo* models of acute myeloid leukemia	(125)
CAR 5/IL15-transduced CB-NK cells	CD5 CAR engineered IL15-transduced NK cells	Ongoing phase I/II: for the management of relapsed/refractory hematological malignancies	NCT05110742
CD4-IL15/ IL15sushi CAR T cells	Modified CD4 CAR to secrete an IL-15/IL-15sushi complex	Anti-T-cell malignancies effect *in vitro, in vivo* and in a phase I trial with 3 patients, who achieved complete response	(126)
mbIL15-CAR T cells	CAR coexpresses a membrane-bound chimeric IL-15 (mbIL15)	Potent rejection of CD19+ leukemia	(127)
161533 TriKE	Trispecific killer engager containing single chain scFv against CD16 and CD33 and modified human IL15 crosslinker	Restored potent NK function against primary acute myeloid leukemia and induced specific NK cell proliferation at the immunologic synapse between NK and CD33+ cells	(128–130)

This table details the structure and functions of the compound , together with a list of pre-clinical and clinical studies with them.

IL-15 has the highest potential as a therapeutic tool when administered with the purpose of eliciting tumor eradication through the activation of NK cells and T-lymphocytes. Major challenges in employing IL-15 as an anti-tumoral agent are its limiting pharmacokinetics and the risk of toxicities. With an *in vivo* half-life of 2.5 hours and its unstable protein structure, the IL-15 has demonstrated a limited effect, nevertheless safe for infusion therapy in clinical use (91, 131). With a deeper understanding of IL-15 biology, new therapeutic agents have been developed to improve *in vivo* pharmacokinetics, mimic physiologic IL-15:IL-15Rα *trans*-presentation and reduce the risk of toxicity.

The results of preclinical and clinical studies have brought to the conclusion that IL-15 is not enough as a single agent and produces the best effects when in combination with other agents, namely checkpoint inhibitors and monoclonal antibodies (132–139). IL-15 treatment is currently being evaluated in several ongoing clinical studies in conjunction with immune checkpoint blockade (#NCT02523469, #NCT04261439, #NCT03905135), monoclonal antibody (#NCT02689453) and bispecific monoclonal antibodies (#NCT02384954).

The most recent publications on IL-15 concern its use in the field of cellular adoptive therapies, both as an adjuvant in the *in vitro* expansion of CAR-T and CAR-NK cells and in combination with the cellular infusion to propagate and maintain the cellular therapies *in vivo*. In the phase I/II clinical trial of CD19 CAR-T for B-cell malignancies (NCT01865617), low serum IL-15 post-CAR-T infusion concentration was related to inferior CAR-T kinetics and, analogously, high levels of IL-15 in the serum were associated with post-CAR-T cell favorable outcomes, highlighting the importance of this cytokine in the adoptive cell therapy response (140). Novel approaches have integrated the IL-15 gene into the adoptive cells to allow them to secrete the cytokine and auto-sustain their growth, expansion, and cytotoxic function against the tumoral cells (123, 124, 141), with promising clinical activity. The newest engineered cell products are listed in **Table 1**.

## Discussion and conclusions

This minireview aimed to picture a general update on the role of IL-15 in the pathogenesis of hematological malignancies and to briefly describe the use of IL-15-based therapies.

The complex transcriptional and posttranscriptional regulatome of IL-15 represents a major challenge in assessing IL-15 expression in hematological malignancies. There is a need for a systematic evaluation of IL-15 protein expression in B- and T-cell neoplasms, especially considering its eminent role as a combination agent for immunotherapy and adoptive cell therapy.

Of relevance is the association between IL-15-driven malignancies and autoimmunity: LGLL and EATL shared a background of autoimmune diseases and are CD8+ T-cell malignancies. IL-15 differs from IL-2 in its ability to inhibit AICD and have no effect on the Treg subpopulation. Overexpression of IL-15 can be, thus, directly responsible of the persistence of self-reactive lymphocytes that lead to autoimmunity. IL-15 also creates a microenvironment of chronic inflammation, facilitating the malignant transformation of the proliferating lymphocytes. Studying the biology and function of IL-15 can lead to a better understanding of the still unexplored relationship between lymphoproliferative disorders and autoimmunity.

The major trend in the recent literature involves using IL-15 agonists and cytokine-receptor complexes as enhancers of anti-tumor immune response and as combination agents with immunotherapy and adoptive cell therapy. From the results of the most recent clinical trials, the latest option is preferred since the efficacy of IL-15 as monotherapy seems inadequate to induce cancer regression.

However, IL-15 can have a pathogenic role in the development and progression of B- and T-cell neoplasms and different strategies have been established to block its downstream signaling pathways to inhibit malignant cell proliferation. The fact that in hematological malignancies, the tumoral cells are also immune cells can explain this paradoxical role of IL-15. An undetermined aspect is the effect of the administration of exogenous IL-15 for the treatment of known IL-15-driven malignancies: the autocrine and paracrine production of IL-15 from the tumoral cells and the tumoral microenvironment can have opposite effects compared to the exogenous cytokine, and it can be even curative through the inhibition of the IL-15 endogenous production. Further studies are needed, with the aim of avoiding disease progression and systemic toxicities. In this context, the use of a standardized laboratory test to monitor the levels of IL-15 before, during and after IL-15-based therapies is of paramount importance. In addition to this, factors such as the technical and biological variables that affect the commutability of the IL-15 should also be considered. Different levels of IL-15 should be measured between different groups of patients or over time using the same procedure and platform. The data collected from the profiling of IL-15 should be correlated with the results of nucleic acid amplification and imaging observations to obtain an accurate assessment of the disease progression.

A discussed challenge about the use of IL-15 as anti-cancer therapy is the probable effect of chronic exposure of NK cells to IL-15, which can produce a paradoxical reduction in efficacy, due to progressive exhaustion of the target cells, through the cell cycle arrest and decreased cell survival (142). This conclusion is a debate because other groups demonstrated unchanged cell viability and function of NK cells after sustained IL-15 exposure *in vivo* (143). It is also important to note that the effects of systemic administration of IL-15 on autologous or allogeneic NK cells have been explored in AML patients. IL-15 can trigger the expansion of recipient CD8+ T cells, which can accelerate donor NK rejection. Indeed, the use of systemic IL-15 in combination with allogeneic cell therapy could limit the clinical activity and therapeutic window of these therapies. Two independent clinical trials on relapse/refractory acute myeloid leukemia cohorts treated with NK cell therapy disclosed a lower clinical activity when systemic IL-15 (IL-15; N-803) was used for support, compared with IL-2 (144).

In summary, harnessing IL-15 as an anti-cancer drug should be considered with caution and its appropriateness for a particular type of cancer, especially hematological malignancies, must be carefully assessed.

## Author contributions

PS, HP, and AM planned and conceptualized the review. PS and HP wrote the initial draft and have contributed equally. PS, HP, CI, AM, JB, NC, and PP contributed to writing, reviewing, and revision. All authors contributed to the article and approved the submitted version.
